# Recent advances in optical fiber-based gas sensors utilizing light-induced acoustic/elastic techniques

**DOI:** 10.1016/j.pacs.2025.100715

**Published:** 2025-03-28

**Authors:** Yuhui Liu, Yue Qi, Yangjian Cai, Xiaoyi Bao, Song Gao

**Affiliations:** a Shandong Provincial Engineering and Technical Center of Light Manipulations & Shandong Provincial Key Laboratory of Optics and Photonic Device, School of Physics and Electronics, Shandong Normal University, Jinan 250014, China; b University of Ottawa, Department of Physics, Ottawa (ON), K1N 6N5, Canada

**Keywords:** Gas sensor, Optical fiber sensor, Brillouin scattering, Spectroscopy

## Abstract

Gas sensing detects gas properties, such as physical, molecular, optical, thermodynamic, and dynamic properties. Light-induced acoustic techniques include monitoring the optical and physical properties of the gas. Fiber-based gas sensing is important because it offers several unique advantages compared to traditional gas sensing technologies, such as high sensitivity and accuracy, a compact and lightweight design, remote sensing capabilities, multiplexing, and distributed sensing. We review the recent developments in optical fiber-based gas sensors utilizing light-induced acoustic/elastic techniques based on photoacoustic spectroscopy, Brillouin scattering, and light-induced thermoelastic spectroscopy (LITES).

## 1 Introduction

Gas sensing involves detecting and measuring the presence or concentration of gases using technologies like optical fibers, semiconductors, or chemical sensors. They hold a pivotal role in both daily life and industrial production [1] and have been widely used in fields such as environmental monitoring [2], [3], home life [4], aerospace [5], petroleum exploration [6] and human health monitoring [7]. Various types of gas sensors with distinguished gas-sensing mechanisms have been proposed such as electrochemical gas sensors [8], contact combustion based gas sensors [9], optical gas sensors [10], and semiconductor-based gas sensors [11]. Among them, optical gas sensors have the advantages of high selectivity, high sensitivity, fast response speed, less cross interference, and in-situ detection capability [12]. Over the past decades, several optical gas-sensing mechanisms have been reported, such as absorption spectroscopy [13], and photoacoustic effect [14], [15], [16]. In gas sensing via absorption spectroscopy, a specific wavelength of light that is matched with gas absorption spectra is emitted and passed through a gas sample. The gas molecules absorb light which results in a reduction in light intensity. By measuring this change in intensity due to absorption, the concentration of the gas can be accurately determined. In gas sensing utilizing the photoacoustic effect, a gas sample is subjected to modulated light, causing the gas molecules to absorb the light energy and subsequently relax non-radiatively through molecular collisions. This absorption process results in a cyclical heating of the gas, which subsequently generates acoustic waves (also known as sound waves). The intensity and frequency of these acoustic waves are indicative of the gas concentration and its absorption characteristics. Among all mechanisms, light-induced acoustic/elastic techniques involve the use of modulated or pulsed light to interact with a material, causing localized heating or deformation due to energy absorption. This interaction results in the generation of acoustic waves (sound) or elastic waves (mechanical vibrations), which can be analyzed to ascertain the material’s physical or chemical properties, such as gas concentration or structural characteristics. The frequency modulation employed in these techniques is selected to optimize the production of acoustic or elastic waves, taking into account the material’s properties, resonance effects, and the sensitivity of the detection system. The fundamental principle underlying light-induced acoustic/elastic techniques revolves around the photothermal effect [17], photoelastic effects [18], and material nonlinearity [19].

Gas sensing based fiber optics has been widely studied due to its high detection sensitivity, resistance to electromagnetic interference, fast detection speed, and portability [20]. The incorporation of fiber optical components, serving as substitutes for free space optical elements like lenses, polarizers, and reflective mirrors, offers notable advantages. It fosters a more compact design and enhances compatibility for multi-point detection systems. Furthermore, this transition minimizes the vulnerability to interference and optical component alignment inaccuracies [21]. We will summarize the fiber types used in gas sensing and present an overview of mainstream fiber-based gas sensors utilizing light-induced acoustic/elastic techniques, focusing specifically on three distinct techniques: photoacoustic spectroscopy, Brillouin scattering, and light-induced thermoelastic spectroscopy.

**Fig. 1:**
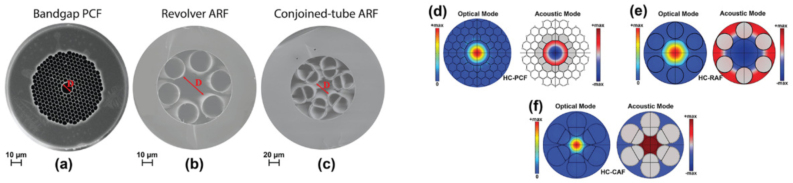
Hollow-core optical fibers with D as the core diameter: (a) HC-PCF; (b) HC-RAF; and (c) HC-CAF. Fundamental optical modes along with the fundamental acoustic modes for the fibers: (d) HC-PCF; (e) HC-RAF; and (f) HC-CAF [22].

**Fig. 2:**
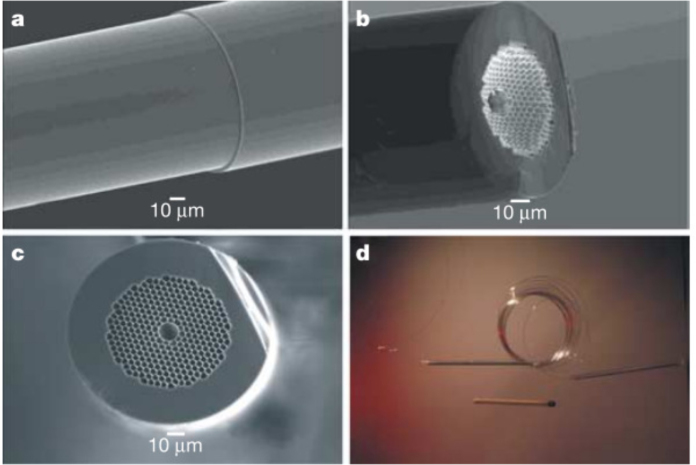
HC-PCF-based gas-cell assembly [23].

## 2 Fibers utilized in gas sensing

In fiber sensing, the interaction between gas and optical fiber is crucial for detecting gas properties. Prior to the incorporation of optical fibers in gas detection, previous measurement systems were hampered by beam divergence, optical losses, and size constraints stemming from the reliance on free space optics. With the development of fiber optical technology, fiber optical sensors have gradually become an important type in gas sensing applications due to the advantages of corrosion resistance, electromagnetic interference resistance, and long-distance and low-loss signal transmission [24], [25]. Due to their distinctive structures and properties, hollow-core fibers [26], [27], photonic crystal fibers with their cladding region featuring a periodic air hole array [28], micro/nano fibers [29] and specialty fiber structures have emerged as prevalent choices in gas sensor research among the diverse range of optical fibers.

### 2.1 Hollow-core fibers (HCFs)

The primary distinction between the hollow-core fibers (HCFs) and the traditional fibers lies in their hollow core, primarily occupied by gas, enabling light-gas interaction within the fiber core with exceptional mechanical stability and flexibility in the development of gas sensors [30]. Fig. 1 shows scanning electron microscope (SEM) images of different types of HCFs and the optical and acoustic modes in the cross sections. There are mainly three types of HCFs, including hollow-core photonic crystal fibers (HC-PCFs), hollow-core photonic bandgap fibers (HC-PBFs) and hollow-core anti-resonant fibers (HC-ARFs).

In 1999, R.F. Cregan et al. reported the principle of fabricating hollow-core photonic crystal fibers (HC-PCFs) [33]. The advancement of HC-PCF is fueled by the exploration of novel guiding mechanisms, such as photonic bandgap (PBG) or inhibited coupling (IC), which stem from quantum mechanics and solid-state physics, rather than traditional optical principles that depend on total reflection [34]. In 2002, F. Benabid et al. introduced a low-loss HC-PCF [35]. Over the past two decades, researchers have implemented numerous innovations in the manufacturing and design of HC-PCFs to minimize losses [36], [37]. HC-PCF has emerged as a pivotal component in the gas sensing field due to its advantages such as high signal-to-noise ratio spectral features derived from the long interaction length and small modal area of optical fibers [34]. Light and gas media are confined within the microscale core of PCF, enabling highly effective photo-gas interactions.

**Fig. 3:**
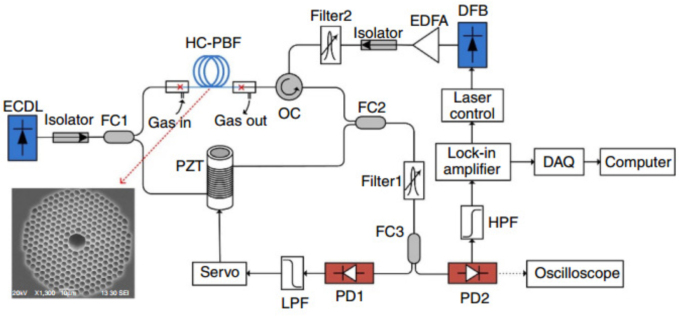
Experimental set-up for gas detection with 10 m long HC-PBF [31].

**Fig. 4:**
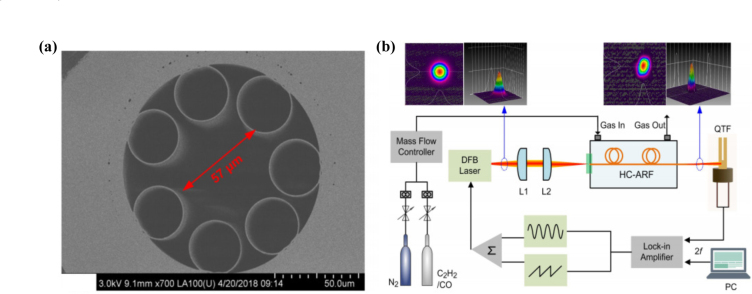
(a) Cross section of HC-ARF; (b) Schematic of the sensor setup for hollow-core anti-resonant fiber based light-induced thermoelastic spectroscopy gas sensing [32].

In 2005, F. Benabid et al. proposed the creation of compact, stable, and highly efficient all-fiber gas units that utilize hollow photonic crystal fibers (HC-PCF) [23]. These units are capable of guiding light over extended distances while exhibiting exceptional performance, stability, and user-friendliness. As depicted in Fig. 2, the all-fiber gas cell comprises a hollow photonic crystal fiber (HC-PCF) filled with gas, sealed securely at both ends, and spliced onto a standard single-mode fiber (SMF). The photonic bandgap created within the “photonic crystal” cladding guides the light in a single transverse mode. Concurrently, the introduction of the all-fiber gas cell established the groundwork for utilizing hollow-core fibers in gas detection applications.

In 2007, A. M. Cubillas et al. showcased the application of hollow-core photonic bandgap fibers (HC-PBFs) for methane gas detection within the 1670nm absorption band [38]. The extended interaction length afforded by HC-PBFs enables sensitive detection of weakly absorbing gases such as methane, attributed to the extensive optical path achievable through HC-PBF technology. Drawing upon the benefits of hollow-core fibers, W. Jin et al. introduced an innovative all-fiber photothermal interference system in 2015 [31]. This system employs hollow-core photonic bandgap fibers (HC-PBFs) to attain ultra-high sensitivity for trace gas detection across a broad dynamic range. As illustrated in Fig. 3, the sensing arm of the fiber-optic Mach–Zehnder interferometer (MZI) comprises a gas-filled hollow-core photonic bandgap fiber (HC-PBF), while the reference arm consists of a standard single-mode fiber (SMF) wrapped around a piezoelectric transducer (PZT). When gas absorption induces phase shifts in the HC-PBF, these changes result in corresponding variations in light intensity at the interferometer’s output. In 2016, F. Yang et al. proposed a sensitive and compact gas sensor based on a hollow photonic bandgap fiber (HC-PBF) [39]. The sensor also uses a hollow photonic bandgap fiber (HC-PBF) with a length of 2 cm, which is fused to a single-mode fiber tail at both ends and has holes on the side for gas filling. The absorption of the modulated pump beam within the hollow-core leads to phase modulation of the detection beam, which is subsequently detected by a Fabry–Perot interferometer (FPI).

In order to further improve the detection performance of fiber-based gas sensors and minimize mode interference noise, hollow-core anti-resonant fibers (HC-ARFs) were proposed as light medium and gas cell at the same time [32], [40]. Fig. 4(a) shows the image of the cross section of HC-ARF and Fig. 4(b) presents the measurement setup [32]. HC-ARFs boast remarkable benefits, including near-single-mode transmission, minimal optical loss, and a broad spectral range. Furthermore, their innovative design—featuring a ring of silica capillaries surrounding a hollow core—efficiently mitigates mode interference between the core mode and other cladding modes [28]. Conversely, HC-ARFs incorporate a minute hollow core in their central area, which not only facilitates the propagation of light modes but also concurrently confines gas samples within the hollow core. The sensor utilizing HC-ARFs demonstrates an outstanding linear response to analyte concentration, achieving a minimum detection limit (MDL) of 4.75 ppm for $C_{2}H_{2}$ and 1704 ppm for CO, respectively.

**Fig. 5:**
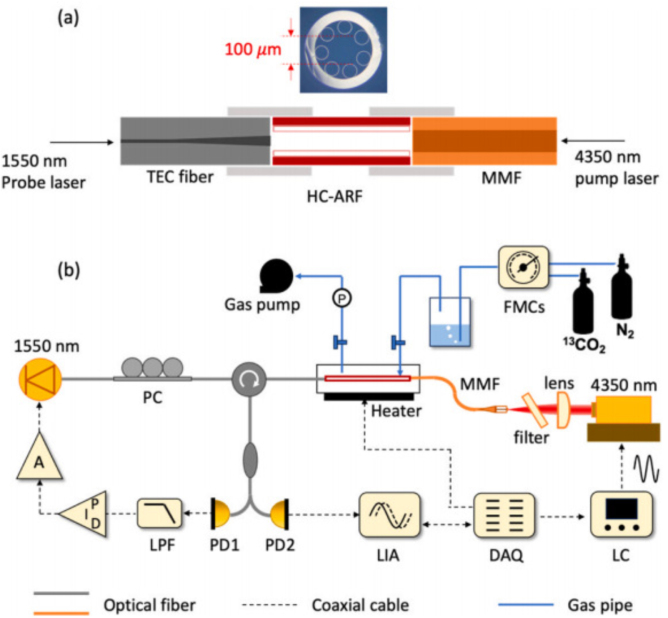
(a) Schematic diagram of the HC-ARF F-P cavity. (b) Experimental setup of the PTI gas sensing of ${}^{13}{\mathrm{CO}}_{2}$[41].

In 2024, C. Yao et al. also used Fabry–Perot photothermal interferometry (PTI) technology with hollow-core anti-resonant fibers (HC-ARFs) for sensitive gas detection in the mid infrared region [41]. A HC-ARF serves as both a light guide and a gas cell, achieving a compact sensor design. The authors presented a PTI design by splicing the HC-ARF between solid fibers to form an F-P cavity, making the PTI very short, as shown in Fig. 5(a). Thus, the mid-infrared pump beam was effectively coupled into the HC-ARF, successfully increasing the pump power by 6.7 times. The sensor was used to detect ${}^{13}{\mathrm{CO}}_{2}$ at 4.35 μm and achieved a detection limit of 0.4 ppb of ${}^{13}{\mathrm{CO}}_{2}$ within 240 s of integration time.

### 2.2 Photonic crystal fibers (PCFs)

Photonic crystal fibers (PCFs), characterized by a periodic array of air holes in their cladding region, represent another significant advancement in gas sensing [28]. The incorporation of air holes within the silica cladding area offers novel possibilities for exploiting the interaction between light and gas through the evanescent field present within these holes [42]. By optimizing device structural parameters, such as hole size, PCF-based gas sensors exhibit high sensitivity and remarkable manufacturing flexibility [43], [44], [45]. Additionally, PCF exhibits characteristics such as single mode guidance [46], controlled dispersion [47], [48], a large effective mode area [49], high birefringence [50], low Fresnel reflection, and minimal loss [51]. The distinctive properties of PCFs pave the way for overcoming challenges like complex sensor structures, high costs, and limited sensitivity inherent in traditional sensors.

### 2.3 Micro-nano fibers

**Fig. 6:**
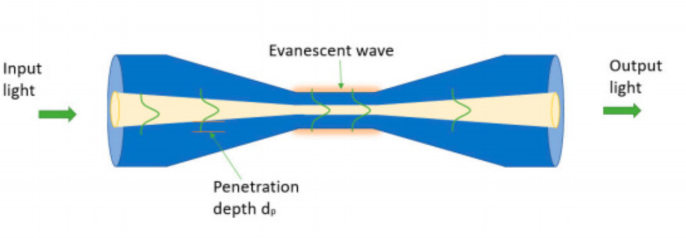
Schematic diagram of the taper waist structure of the Micro-nano fiber [58].

Micro-nano fibers are a type of waveguide with a diameter close to or smaller than the wavelength of the transmitted light. When the diameter of the fiber core is close to or below the guiding wavelength, micro-nano fibers exhibit a variety of unique waveguide characteristics in sensing applications [1], [29], [52], [53], [54]. Utilizing flame, laser, or electric heating to stretch glass optical fibers, micro-nano optical fibers can be prepared through physical stretching methods [55], [56]. As the diameter of a fiber is reduced to the wavelength scale, a substantial amount of the optical mode power resides outside the micro-nano fibers, offering potential utilization for gas detection purposes [57]. Micro-nano fiber gas sensors offer distinct advantages, including rapid response speed, high sensitivity, and low power consumption. In 2023, K. A. Stasiewicz et al. coated tapered optical fibers with graphene oxide layers, which realized the rapid detection of different gases, including nitrogen, hydrogen, and a propane-butane mixture [58]. Fig. 6 shows the structure of the micro-nano fiber. Light is transmitted through the tapered region, with the penetration depth ($d_{p}$) serving to characterize the portion of the light guided within the optical fiber core that extends into the cladding as the evanescent field.

### 2.4 Specialty fiber structure

**Fig. 7:**
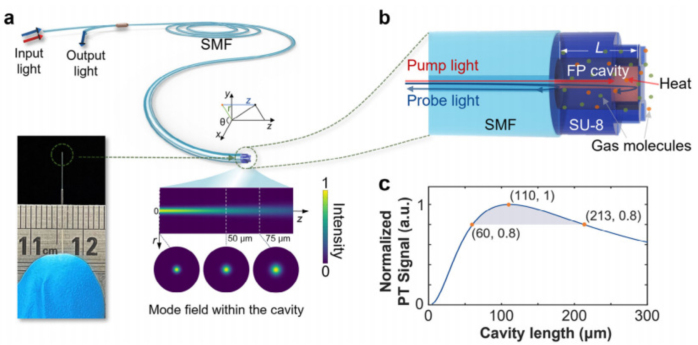
(a) Schematic of optical fiber-tip PTI gas microsensor. (b) Working principle of FP cavity-based PT interferometry. (c) Calculated dependence of PT signal on the cavity length L when the RI modulation frequency is 21 kHz [59].

Currently, state-of-the-art fiber optical gas sensors often employ lengthy fibers as gas absorption components to enhance their sensitivity in detecting gases. However, they confront obstacles such as sluggish response times, inadequate selectivity, and restricted applicability in confined areas. In April 2024, P. Zhao et al. proposed an optical fiber-tip gas sensor, which has been fabricated by directly 3D micro-printing a Fabry–Perot (FP) cavity onto the end face of a standard single-mode optical fiber [59]. The microcavity is only 66 μm in length, as shown in Fig. 7. The laser absorption by gas molecules induced a change of temperature (Refractive index) in the cavity, as marked in red. When the pump beam propagates through the cavity, it gets absorbed by $C_{2}H_{2}$ molecules, leading to a periodic heating of the gas sample through non-radiative relaxation. Consequently, this process modulates the refractive index (RI) of the sample. This modulation in RI, in turn, triggers a corresponding modulation in the phase of the probe beam that is reflected off the suspended thin film of the Fabry–Perot (FP) cavity. The sensor can detect $C_{2}H_{2}$ gas concentration of 160 ppb with a response time of only 0.5 s. This method directly manufactures F-P chambers on the fiber end face, which forms a key component of the fiber tip photothermal interference gas sensor, making the fiber tip microsensor both ultra compact and highly durable, suitable for space limited applications.

**Fig. 8:**
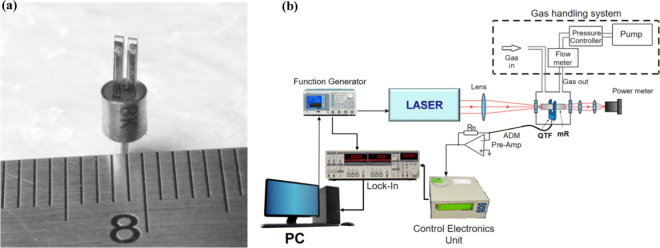
(a) Photograph of a typical wristwatch TF; (b) Schematic of a typical QEPAS sensor setup [60].

## 3 Optical fiber gas sensors utilizing light-induced acoustic/elastic techniques

### 3.1 Photoacoustic gas sensors

Photoacoustic gas sensors have been extensively investigated with a simple and low-cost structure, good selectivity and high sensitivity. As far back as the late 19th century, scientists had elucidated the photoacoustic effect [61], [62]. To address the limitations of cross-sensitivity and inadequate anti-interference capabilities in gas absorption spectroscopy-based non-dispersive infrared gas sensors, research into gas sensing utilizing the photoacoustic effect has unveiled significant potential [63], [64]. Photoacoustic spectroscopy (PAS) is a potent analytical method that integrates the principles of light absorption and sound generation to investigate the optical and acoustic characteristics of materials. The fundamental principle of PAS hinges on the photoacoustic effect, where the sample is illuminated with modulated light, resulting in the generation of weak sound waves through photoacoustic excitation within the sample gas. Therefore, sound detectors, rather than optical detectors, are utilized, as they demonstrate insensitivity to the wavelength of the light source. When modulated light is shone onto a gas sample, the gas molecules absorb the light energy and undergo transitions from their ground state to an excited state. As these molecules relax back to their ground state, they release heat, causing rapid and localized temperature changes within the gas sample [65]. These temperature fluctuations lead to pressure waves, which manifest as sound waves. These sound waves are then detected by a sensitive acoustic detector, such as a microphone or a quartz tuning fork, and converted into an electrical signal for analysis. By measuring the amplitude and phase of the acoustic signal as a function of modulation frequency, information on the light absorption characteristics of the sample can be obtained [66]. The field has witnessed significant advancements with numerous new applications and technological innovations based on photoacoustic spectroscopy being introduced recently. In 2024, G. Wu et al. introduced a single fiber-type double cavity enhanced photoacoustic spectroscopy sensor designed for trace methane detection, achieving a detection limit of 1.13 ppm with an integration time of 100 s [67]. That same year, K. Kinjalk et al. developed a quartz-enhanced photoacoustic spectroscopy system for highly selective and sensitive detection of volatile organic compounds, achieving minimum detection limits of 113 ppb for toluene, 3 ppb for benzene, and 3 ppm for propane [68]. Meanwhile, J. Wang et al. proposed an innovative quartz-enhanced multiheterodyne resonant photoacoustic spectroscopy technique. By employing a quartz tuning fork as a high-Q sound transducer and integrating it with a phase-sensitive detector, the method extracts resonant sound components from multiple heterodyne acoustic tones, offering a simple and cost-effective hardware solution [69]. This approach enables wavelength-independent DCS detection with a noise equivalent absorption of $5.99\times 10^{-6}\;{\mathrm{cm}}^{-1}\;{\mathrm{Hz}}^{-1/2}$.

#### 3.1.1 Fiber-based QEPAS

In 2002, researchers initially suggested utilizing a quartz tuning fork (QTF) as an acoustic transducer for the detection of trace gases through photoacoustic methods, a technology referred to as quartz-enhanced photoacoustic spectroscopy (QEPAS) [70]. A QTF is employed to detect the sound waves that are produced by the non-radiative relaxation of energy within target gas molecules, which are excited by a modulated laser source [21]. Fig. 8(a) shows the photograph of a typical wristwatch tuning fork.

The structure of a typical QEPAS sensor setup is shown in Fig. 8(b). The laser beam is directed and focused precisely between the two sharp prongs of QTF in order to stimulate the acousto-optic effect and elicit vibrations within the QTF. Owing to the exceptionally high quality factor of the quartz tuning fork (Q > 10,000 at standard atmospheric pressure), sound energy can efficiently accumulate within its robust, noise-resistant quadrupole structure. The detected optical signal S can be represented as [60]:

(1) $$S=\frac{k\alpha QPC}{fA}$$

where k is a constant, α is the absorption coefficient of the target gas, C is the concentration of the target gas, P is the laser power, f is the resonance frequency, and A is the resonator cross-section area.

QEPAS sensor systems generally necessitate certain optical components in the open path to enhance beam quality and mitigate the production of noise. Nonetheless, the incorporation of optical lenses can pose challenges, including an increase in sensor size and complexities in assembly. Additionally, the adjustment of the lenses’ relative position, as well as their radial and axial alignment, is necessary, which to some extent constrains the practical deployment of the sensors [71].

**Fig. 9:**
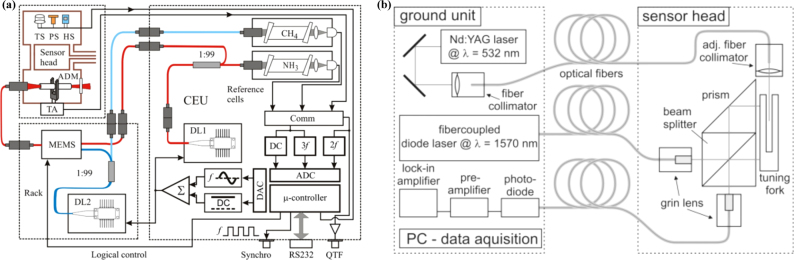
(a) Schematic of a compact two-gas QEPAS sensor [73]; (b) Schematic drawing of the all-fiber-coupled ozone sensor experimental setup [74].

Fiber-based QEPAS represents a quintessential gas detection technology, seamlessly integrating the exceptional sensitivity of QEPAS with the robust anti-interference capabilities of optical fibers. Additionally, it boasts a straightforward structure and facilitates the formation of sensing networks with remarkable ease [72]. In May 2012, L. Dong et al. employed a fiber coupled QEPAS acoustic detection module to accomplish quantitative analysis of trace amounts of ${\mathrm{CH}}_{4}$ and ${\mathrm{NH}}_{3}$ in impure $H_{2}$ [73]. This sensor achieves dual gas detection by utilizing two distinct diode lasers with different wavelengths, each targeting the respective gas absorption line, thereby enabling highly sensitive detection within the near-infrared spectral band. The experimental setup is depicted in Fig. 9(a). Despite being partially structurally coupled with optical fibers, the components of the sensor system are neatly integrated thanks to the inherent structural advantages of optical fibers. In August 2012, M. Kohring et al. proposed an all-fiber-coupled ozone sensor, leveraging tuning fork-enhanced photoacoustic spectroscopy, boasting a detection limit of S=2.13±0.02 ppm [74]. The sensor structure is shown in Fig. 9(b). The ground unit and the sensor head are interconnected solely through a single-mode optical fiber. By conveying laser sources via optical fibers, the QEPAS sensor systems can be enhanced in terms of their versatility for flexible laser beam guidance and compact design.

**Fig. 10:**
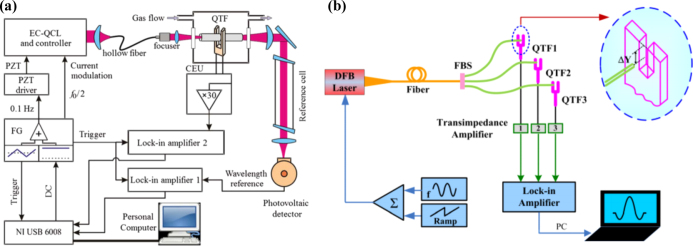
(a) Schematic of QCL hollow-core-coupled QEPAS sensor [75]; (b) Schematic configuration of an all-fiber QEPAS sensor system [76].

Due to the significant absorption characteristics of fiber optic silica materials in the mid-infrared region, the transmitted light experiences heightened attenuation, constraining the performance of mid-infrared QEPAS sensors that rely on single-mode fiber transmission [72], [77]. Utilizing the benefits of hollow-core fibers, the beam can sustain single-mode transmission at mid-infrared wavelengths while experiencing minimal transmission loss. In November 2012, V. Spagnolo et al. introduced a sensor that utilizes hollow-core fiber technology coupled with a quantum cascade laser (QCL) at a wavelength of 10.54 μm and quartz-enhanced photoacoustic spectroscopy (QEPAS) [75]. Fig. 10(a) shows the experimental setup. A collimating optical system was meticulously designed to produce a laser beam featuring a notably diminished beam diameter and waist circumference. The design and implementation of the mid-infrared fiber, in conjunction with the collimating optical system, guarantee the seamless transmission of single-mode QCL beams to QEPAS sensors. With a QCL power of 18 mW, the system achieved a remarkable minimum detection sensitivity of 50 ppt for ${\mathrm{SF}}_{6}$ gas.

After conducting extensive research on the integration of optical fibers and QEPAS technology, Y. Ma et al. constructed an ultra-compact, all-fiber quartz-enhanced photoacoustic spectroscopy (QEPAS) sensor in 2016 with the experimental setup shown in Fig. 10(b) [76]. In comparison to traditional QEPAS sensors, this sensor boasts numerous advantages, including easier optical alignment, reduced insertion loss, lower cost, and a more compact design. Furthermore, it showcases the potential for spatial resolution measurement by utilizing fiber optic splitters and three QTFs for multi-point detection. In the same year, Z. Li et al. proposed the utilization of multimode fiber (MMF) instead of single-mode fibers in the mid-infrared band for the construction of QEPAS sensing systems [78]. Sensitive detection of nitric oxide (NO) was conducted at a wavelength close to 5.26 μm. The findings reveal that the sensor exhibits minimal sensitivity to fiber bending noise when the bending radius is maintained at 5 cm. Furthermore, the minimum detectable limit (MDL) for NO gas can achieve a remarkable level of 24 ppb.

**Fig. 11:**
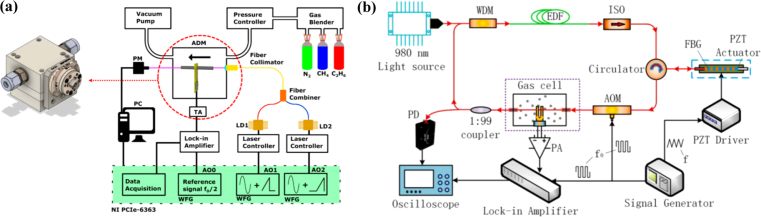
(a) Schematic of the Fiber-Coupled QEPAS experimental apparatus [79]; (b) Configuration of the novel wavelength scanning Q-switched FLI-QEPAS $C_{2}H_{2}$ sensing system [80].

In 2020, G. Menduni et al. proposed a dual gas detection system based on fiber coupled QEPAS achieving continuous detection of methane and ethane in the near-infrared range [79]. They designed and implemented an all-fiber structure, depicted in Fig. 11(a), which utilizes two distinct laser wavelengths (1653.7nm and 1684nm) for the detection of methane and ethane, respectively. In 2021, F. Wang et al. reported a novel fiber ring laser cavity quartz enhanced photoacoustic spectroscopy (FLI-QEPAS) using wavelength scanning Q-switching technology for acetylene detection [80]. The system employs an erbium-doped fiber as the gain medium for laser generation and incorporates a customized fiber Bragg grating inside the fiber ring cavity, which is connected to a piezoelectric transducer for precise wavelength scanning. This scanning covers the 1531.58 nm absorption line of acetylene. The system’s minimum detection limit can be enhanced to 2.8 ppbv, and its linear dynamic range can extend up to 10000 ppmv. This device represents a groundbreaking innovation by introducing wavelength scanning Q-switching technology into the FLI-QEPPAS system for the first time.

**Fig. 12:**
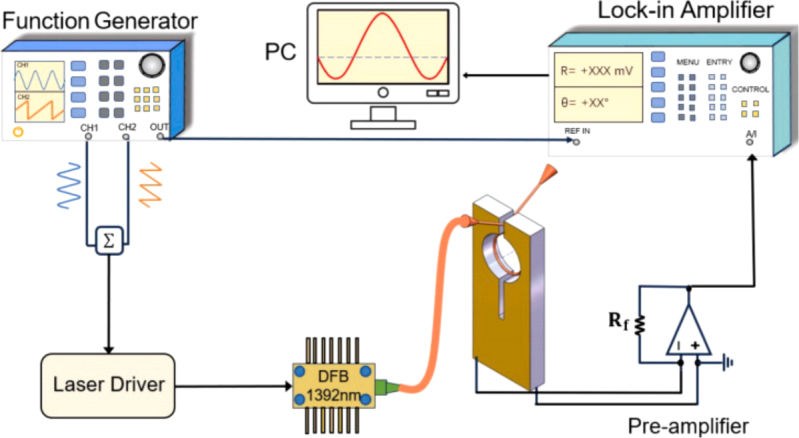
Schematic diagram of the MKR-QEPAS experimental setup [81].

In 2023, J. Xie et al. proposed the microfiber knot resonator enhanced QEPAS [81]. By inducing constructive interference between the microfiber knot resonator and the excitation laser wavelength targeted at $H_{2}O$ molecules, the microfiber knot resonator was integrated with a clamp-type tuning fork. This integration resulted in a significant enhancement of the evanescent resonance, ultimately improving the detection sensitivity by approximately one order of magnitude. The experimental setup was presented in Fig. 12.

**Fig. 13:**
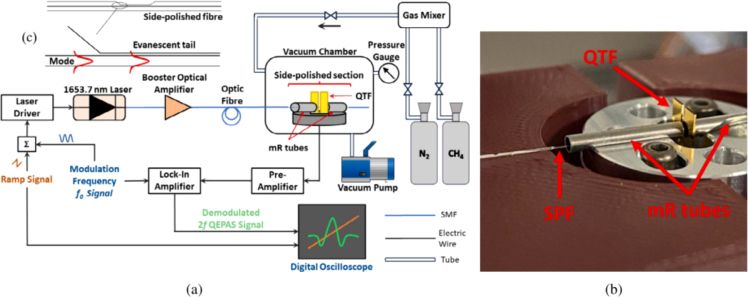
(a) Schematic of EW-QEPAS setup, (b) Image of spectrophone and SPF mount, (c) schematic of an SPF and side-polished region schematically showing mode propagation and access to the evanescent tail of the mode. All light coupling from the laser to the side-polished fiber was done using optical fibers [21].

In 2024, C. Twomey et al. designed an all-fiber laser gas analyzer using side polished fiber optics and QEPAS technology to detect methane [21]. Shown in Fig. 13, the polished face of the side-polished fiber is strategically positioned to allow the evanescent wave to generate a photoacoustic wave, thereby exciting the fundamental flexural mode of the QTF. Consequently, a minimum detection limit of 34 ppmv was determined. This sensor, devoid of any free-space optical components, is an excellent candidate for miniaturization and is well-suited for applications in harsh environments as well as for gas detection tasks that demand maneuverability.

In recent years, optical fiber-based QEPAS gas sensors have achieved remarkable advancements, benefiting from the multifaceted advantages of optical fibers and QTFs. Research and applications of QEPAS utilizing hollow-core fibers, multimode fibers, Q-switched fiber-ring laser and microfiber knot resonator have been explored. In summary, the integration of fiber optic technology with QEPAS technology is propelling the continuous evolution of gas sensors towards higher sensitivity, scalability, stability, and ease of integration and miniaturization, gradually paving the way for novel breakthroughs in the field [72].

#### 3.1.2 Fiber-based CEPAS

**Fig. 14:**
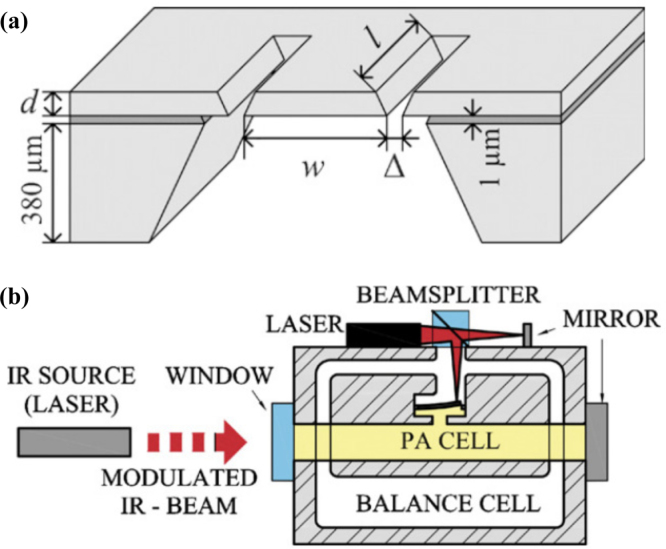
(a) Silicon cantilever sensor. (b) Setup for measurements with a cantilever enhanced PA detector [83].

Cantilever-enhanced photoacoustic spectroscopy (CEPAS) was first introduced by J. Kauppinen et al. in 2004, with the aim of achieving high sensitivity in gas analysis through photoacoustic detection [82]. Traditional photoacoustic spectroscopy employs a microphone as the acoustic wave detector, while CEPAS innovatively utilizes a cantilever as the sensing element. When the acoustic waves generated by the photoacoustic effect act on the cantilever, they induce mechanical vibrations. These vibrations can be captured by high-precision optical detection systems (such as laser interferometers or beam reflection devices) and converted into corresponding electrical signals. Fig. 14(a) and (b) show typical silicon cantilever and CEPAS sensor setup, respectively [83]. Benefiting from the excellent mechanical sensitivity and unique resonance characteristics of the cantilever, the system can detect extremely weak photoacoustic signals. By precisely adjusting the resonance frequency of the cantilever, the response to acoustic signals can be optimized. The use of optical interference techniques, such as laser interferometers, to detect the micro-vibrations of the cantilever can significantly enhance the system’s the detection limit by approximately 100 times [82].

**Fig. 15:**
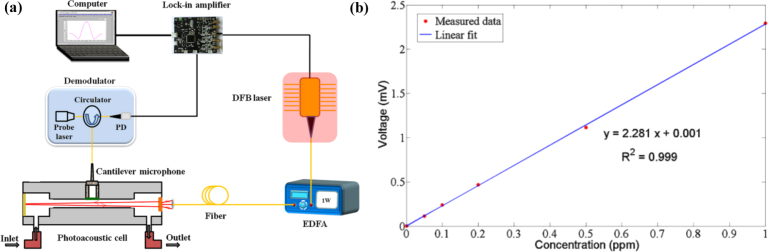
(a) Schematic diagram of the fiber-based CERPAS system. (b) Photoacoustic signals with different concentrations of $C_{2}H_{2}$[84].

However, the significant size of optical cantilever sensors poses challenges in achieving compatibility with PA cells. Furthermore, when the system is in a non-resonant state, the signal-to-noise ratio (SNR) diminishes considerably, thereby constraining the sensitivity of gas detection [83]. In 2018, K. Chen et al. introduced a fiber-based cantilever enhanced resonant photoacoustic spectroscopy (CERPAS) for detecting trace gases by integrating a highly sensitive fiber Fabry–Perot cantilever microphone with resonant PAS technology. This approach leverages the exceptional sensitivity and miniature size of the fiber tip Fabry–Perot interferometer (FPI) [84]. Fig. 15(a) shows the structural diagram of the fiber-based CERPAS system. The tunable distributed feedback (DFB) laser generates a light source centered at a wavelength of 1532.83nm. Following amplification by an erbium-doped fiber amplifier (EDFA), the laser beam is collimated and directed towards a first-order longitudinal resonant photoacoustic (PA) unit. Positioned at the heart of the PA unit, the cantilever beam sensor’s probe detects the peak amplitude of the PA signal. Subsequently, the sound sensor detection module translates the variation in Fabry–Perot cavity length into an alternating electrical signal. This signal undergoes amplification by the lock-in amplifier integrated within the field programmable gate array (FPGA) and is then transmitted to the computer terminal for further processing. Fig. 15(b) illustrates the spectral peaks recorded at varying concentrations of $C_{2}H_{2}$. Notably, the gas detection limit of this sensor surpasses that of previous photoacoustic acetylene sensors by at least one order of magnitude. Subsequently, they introduced an ultra-sensitive trace gas detection technique, leveraging tube cantilever beam dual resonance-enhanced fiber photoacoustic spectroscopy (PAS) [85]. This method integrates the amplitude amplification of photoacoustic pressure waves within an acoustic resonant tube with the heightened responsiveness of the cantilever beam to photoacoustic signals, thereby achieving exceptional sensitivity in gas detection. The results demonstrated that, when detecting acetylene, the noise equivalent detection limit was 27 ppt, and the normalized noise equivalent absorption (NNEA) coefficient reached $4.2\times 10^{-10}$ ${\mathrm{cm}}^{-1}\;W\;{\mathrm{Hz}}^{-1/2}$.

**Fig. 16:**
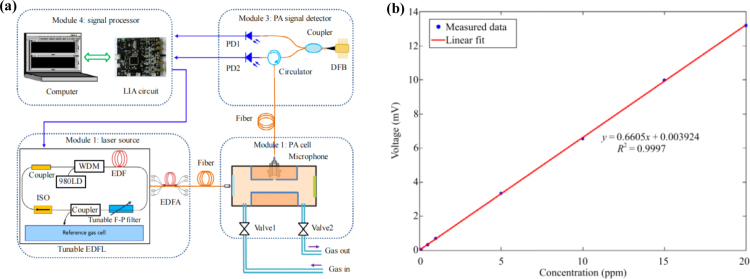
(a) Schematic diagram of the experimental setup of Near-Infrared Fiber-Optic CEPAS; (b) Measured photoacoustic signals of different ${\mathrm{NH}}_{3}$ concentrations [86].

In 2019, M. Guo et al. introduced a near-infrared fiber-optic CEPAS technique designed to detect trace levels of ammonia (${\mathrm{NH}}_{3}$), addressing the challenge of limited sensor sensitivity arising from cantilever beams and PA cells operating in a non-resonant condition [86]. The system structure is shown in Fig. 16(a). This system utilizes a cantilever microphone based on a fiber-optic extrinsic Fabry–Perot interferometer to detect light and sound pressure signals. Using wavelength modulation spectroscopy technology, weak photoacoustic signals are detected. As shown in Fig. 16(b), this fiber-optic CEPAS system exhibits good linear response to trace amounts of ${\mathrm{NH}}_{3}$ in the range of 0 ppm to 20 ppm.

In 2022, M. Guo et al. proposed a compact cantilever-enhanced fiber optic photoacoustic sensor (CEFPS) that is resistant to electromagnetic and environmental noise interference for all-optical passive detection of methane gas in narrow spaces and harsh environments [87]. A photoacoustic cell (PAC), equipped with diffusion holes, has been meticulously designed and constructed. These holes facilitate the circulation of the gas under measurement and effectively mitigate external noise interference within the PAC. The excitation light is introduced into the PAC via a single-mode optical fiber, illuminating the gas within. By demodulating the vibration amplitude of the integrated cantilever beam, which is induced by the photoacoustic pressure, precise concentration information of ${\mathrm{CH}}_{4}$ can be derived.

**Fig. 17:**
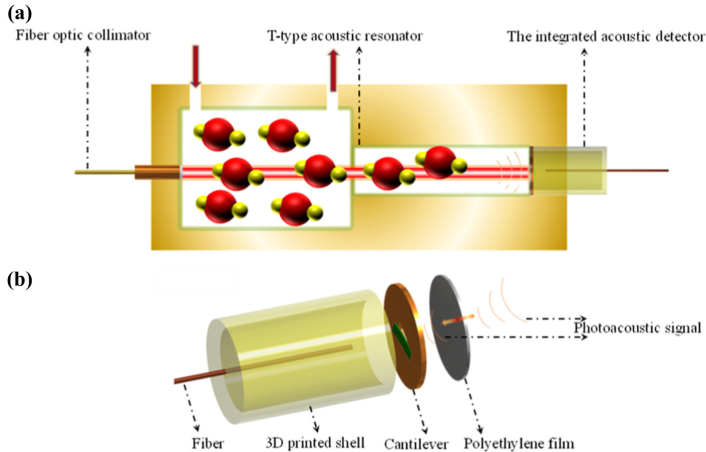
(a) Sketch of the Noncontact Fiber-Optic-CEPAS sensor and (b) enlarged schematic of the integrated acoustic detector [88].

The application of CEPAS in sensing dusty and corrosive gases is constrained due to the necessity for traditional CEPAS measurements to involve direct contact between the cantilever and the gas. In order to overcome this limitation, in 2024, Z. Gong et al. proposed a non-contact fiber-optic CEPAS for high-sensitivity detection and analysis of trace $C_{2}H_{2}$ [88]. Fig. 17(a) shows a schematic diagram of the NCFO-CEPAS sensor. A polyethylene film possessing corrosion-resistant properties is attached to the tip of the cantilever acoustic sensor, and the impact of the spacing between this film and the sensor on the system’s sensitivity is analyzed. Thanks to the elasticity of the polyethylene film, sound energy is transmitted to the silicon cantilever, causing it to vibrate and emit signals as a result of its elastic properties. The experimental measurement results show that the detection limit of $C_{2}H_{2}$ gas is 0.5 ppm, and the normalized noise equivalent absorption coefficient is $5.93\times 10^{-9}$ ${\mathrm{cm}}^{-1}\;W\;{\mathrm{Hz}}^{-1/2}$. Non-contact fiber-optic CEPAS technology boasts not only the benefits of remote, non-contact gas monitoring, immunity to gas corrosion, and robust resistance to electromagnetic interference, but also exhibits a reduced background noise deviation. Additionally, the tested gas never comes into contact with the silicon cantilever beam, thereby preventing any potential damage from corrosive gases to the beam.

**Fig. 18:**
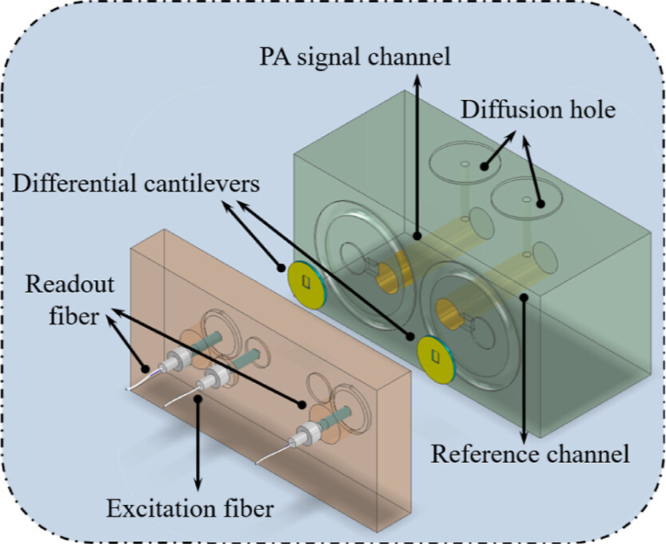
Sketch of the differential-CEPAS sensor [89].

In the same year, to solve the problem of susceptibility of fiber-optic photoacoustic sensors to external vibration and noise interference, C. Li et al. proposed a differential CEPAS for diffusion gas detection [89]. As shown in Fig. 18, the sensor consists of two identically structured PA tubes, a pair of differential interferometric cantilevers, two readout fibers, and a piece of fiber for the transmission of the excitation laser. The laser beam strikes one PA tube through the excitation fiber, serving as the signal channel to stimulate the PA pressure wave. The other tube, devoid of laser incidence, functions as the reference channel to mitigate external disturbances. The differential cantilevers from both channels detect the external interference signals and PA signals contaminated with disturbances. These signals are concurrently restored by a single white-light interferometry demodulator, which utilizes a multiplexed spectral frequency domain of the superimposed interference spectrum. Experimental results indicate that the differential cantilever-enhanced PA sensor exhibits an 80% improvement in ambient noise suppression compared to traditional single-cantilever sensors. This differential fiber-optic CEPAS has good anti-vibration and anti-electromagnetic interference capabilities.

Table 1 summarizes the detection performance of the PA sensors in this review.

**Table 1:** The detection performance of the PA sensors in this review. SMF: single-mode fiber; HCF: hollow-core fiber; SPF: Side-polished fiber; NNEA: normalized noise equivalent absorption coefficient; MDL: minimum detection limit.

Technique	Ref	Target gas	Laser wavelength	Power	Fiber type	NNEA	MDL
			(nm)	(mW)		(cm${}^{-1}$ W Hz${}^{1/2}$)	
QEPAS	[73]	${\mathrm{CH}}_{4}$	1650.95	–	SMF	$2.45\times 10^{-8}$	3.2 ppm
		${\mathrm{NH}}_{3}$	1531.68			$9.1\times 10^{-9}$	1.27 ppm
	[75]	${\mathrm{SF}}_{6}$	10.54	18	HCF	$2.7\times 10^{-10}$	50 ppt
	[76]	$H_{2}O$	1395.07	4.83	SMF	$1.06\times 10^{-7}$	95.4 ppm
	[78]	NO	5.26μm	30	MMF	–	24 ppbv
	[79]	${\mathrm{CH}}_{4}$	1653.7	12	SMF	–	0.76 ppm
		$C_{2}H_{6}$	1684	8.5			34 ppm
	[80]	$C_{2}H_{2}$	1531.58	650	FBG-SMF	–	26 ppbv
	[81]	$H_{2}O$	1.39μm	10	Tapered fiber	$2.97\times 10^{-6}$	–
	[21]	${\mathrm{CH}}_{4}$	1653.7	6.7	SPF	–	34 ppmw

CEPAS	[84]	$C_{2}H_{2}$	1532.83	1000	SMF	–	80 ppt
	[85]	$C_{2}H_{2}$	1532.83	7	SMF	$4.2\times 10^{-10}$	27 ppt
	[86]	${\mathrm{NH}}_{3}$	1522.448	1000	SMF	$2.6\times 10^{-9}$	3.2 ppb
	[87]	${\mathrm{CH}}_{4}$	1650.9	20	SMF	$2.44\times 10^{-8}$	0.32 ppm
	[88]	$C_{2}H_{2}$	1532.8	10.4	SMF	$5.93\times 10^{-9}$	0.5 ppm
	[89]	$C_{2}H_{2}$	1532.83	20	SMF	$1.4\times 10^{-8}$	60 ppb

### 3.2 Brillouin scattering based optical fiber gas sensors

In 1922, L. Brillouin was the first to theoretically propose the concept of inelastic light scattering induced by heat shock waves [90]. This phenomenon was subsequently observed experimentally for the first time by E. Gross in 1930 [91]. Over the past hundred years, the phenomenon of Brillouin scattering has witnessed significant advancements in both theoretical and practical realms. Brillouin scattering is a phenomenon where light interacts with acoustic waves in a medium, such as an optical fiber. When light is incident on the fiber, it can scatter due to the acoustic vibrations within the fiber. These vibrations create local changes in the refractive index of the fiber, leading to the scattering of light. The scattered light carries information about the acoustic waves, which can be detected and analyzed to infer properties of the medium, including the presence of certain gases. Brillouin scattering-based optical fiber gas sensors fit into light-induced acoustic techniques because they use light to stimulate and detect acoustic waves in optical fibers, which are then related to the presence or concentration of gases. Due to the unique properties of Brillouin scattering, such as narrow linewidth and large frequency selectivity gain, the application of this technique spans a variety of scales from microscopy to remote sensing, demonstrating the versatility and potential of Brillouin scattering for gas detection applications [92].

#### 3.2.1 Forward Brillouin scattering

**Fig. 19:**
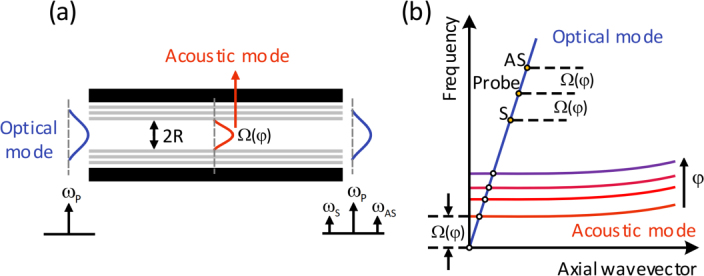
The principle of forward Brillouin scattering for gas concentration detection [16].

Forward Brillouin scattering, also known as Guided Acoustic Wave Brillouin Scattering (GAWBS), is a type of Brillouin scattering that occurs in optical fibers. It is characterized by the co-directional propagation of the pump light and the scattered light [93]. Forward Brillouin scattering involves the interaction between light and acoustic waves in optical fibers. When a pump light wave travels through the fiber, it can interact with the stimulated acoustic waves, leading to the scattering of light [92]. The frequency shift and bandwidth of forward Brillouin scattering is particularly sensitive to changes in fiber structure and environmental conditions. This sensitivity makes it an important tool for characterizing the acoustic modes in optical fibers [94], [95]. Forward stimulated Brillouin scattering, as a strong light-acoustic effect, has applications in material identification, fiber diameter measurement, and high-spatial-resolution sensing [96]. The diagram illustrating the forward Brillouin scattering scheme for gas concentration detection is depicted in Fig. 19. An optical pump light excites an acoustic mode, which subsequently interacts with a probe light traveling along the same waveguide. This interaction results in the generation of a Stokes and an anti-Stokes sideband, each experiencing a frequency shift equivalent to the acoustic resonance frequency Ω. Fig. 19(b) exhibits the acoustic dispersion curve for varying volume concentrations φ. Notably, the acoustic mode within the waveguide is predominantly transverse, allowing the acoustic resonance frequency to be approximated by the cutoff frequency, given by Ω=Cv/R. Here, C represents a constant, v signifies the sound velocity in the gas, and R denotes the core radius of the waveguide. It is worth mentioning that the sound velocity of gases exhibits a linear relationship with the concentration φ. Consequently, the resonant frequency Ω of forward Brillouin scattering serves as a reliable indicator of gas concentration.

**Fig. 20:**
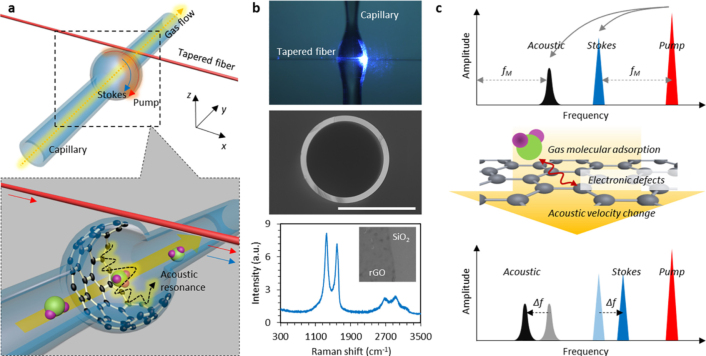
Conceptual design and implementation of the graphene enhanced Brillouin optomechanics [97].

Forward Brillouin scattering is finding increasingly diverse applications in gas sensing. In 2017, B. Yao et al. presented a novel graphene-enhanced Brillouin optomechanical microresonator (GBMR) for ultrasensitive gas detection with a detection limit down to 1 ppb and a wide dynamic range over 5 orders of magnitude for ammonia gas detection [97]. The GBMR consists of a bottle-shaped silica capillary resonator with a layer of reduced graphene oxide (rGO) film deposited on the inner wall. The rGO film enhances the high-order whispering gallery mode (WGM) resonances and enables the forward phase-matched stimulated Brillouin scattering (F-SBS) process, generating high-Q mechanical resonances that are sensitive to gas adsorption on the graphene surface. Shown in Fig. 20(c), the adsorption of gas molecules onto the graphene surface induces changes in local lattice strain, subsequently altering the value of the effective acoustic velocity and, consequently, the phase matching condition. Consequently, the number of gas molecules present on the rGO surface, which directly correlates with the gas concentration within the sensing cell, can be accurately detected. Fig. 20 shows the conceptual design and implementation of the graphene enhanced Brillouin optomechanics. The optical mode is coupled into the microcavity using a tapered fiber with a diameter of about 1 μm that carries the pump light. This tapered fiber can emit and collect the optical mode from the microcavity along the x-axis direction. In this way, the optical mode can enter the microcavity and interact with the acoustic mechanical resonance mode inside the microcavity, producing a confined optical Stokes mode.

**Fig. 21:**
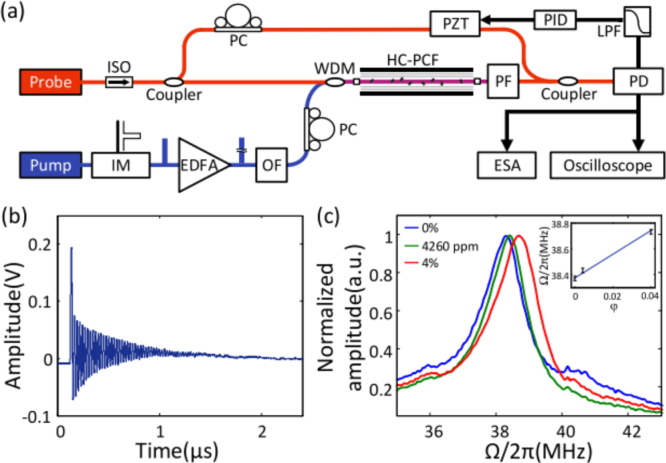
The experimental setup and results of hydrogen detection through FSB in HC-PCFs [16].

In 2018, Y. Zhao et al. achieved detection of hydrogen gas concentration through forward Brillouin scattering (FSB) in hollow-core photonic crystal fibers (HC-PCFs) [16]. FSB is induced by acoustic modes in the nano-scale silica-air matrix and air-filled core of HC-PCFs. The strength and resonant frequency of photon–phonon interaction in FSB are sensitive to gases inside the HC-PCF. As shown in Fig. 21(a), the setup includes a sensing HC-PCF filled with different concentrations of hydrogen balanced with nitrogen, pump and probe beams generated by an erbium-doped fiber amplifier (EDFA), and a Mach–Zehnder (M–Z) interferometer for signal detection. Fig. 21(c) shows the spectra of FBS with different $H_{2}$ concentrations. In addition, the experiment further confirmed that the strength of FBS increases linearly with the total gas pressure within the HC-PCF, whereas the resonant frequency remains largely unaffected by changes in gas pressure.

In recent years, forward Brillouin scattering has attracted considerable attention in the field of substance identification. However, due to issues such as long acoustic wave lifetimes and insufficient signal-to-noise ratios (SNRs), current spatial resolution is limited and falls short of practical requirements. In 2020, C. Pang et al. proposed an optical mechanical time-domain analysis method based on coherent forward stimulated Brillouin scattering detection [98]. The technique employs a two-tone forward stimulated Brillouin scattering probing process, which exhibits stronger interaction intensity and achieves a spatial resolution of 2 m in a 225 m-long optical fiber, which can be utilized for the distributed gas sensing in the future.

**Fig. 22:**
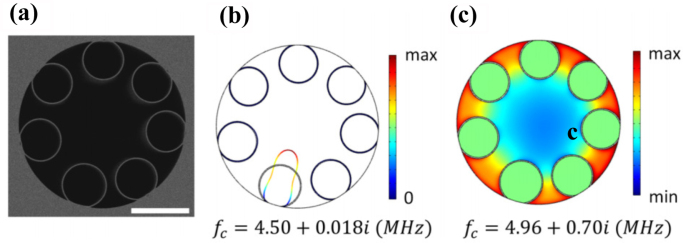
(a) SEM image of the AR-HCF. The white bar is 20μm; (b) A capillary mode ($w_{1}$); (c) An air mode ($R_{01}$) [99].

In 2021, Y. Zhao et al. demonstrated a new approach called photoacoustic Brillouin spectroscopy (PABS) that exploits the light-gas-acoustic interaction in a gas-filled anti-resonant hollow-core fiber (AR-HCF) for high-sensitivity gas sensing and non-invasive fiber characterization [99]. The acoustic modes of the AR-HCF are excited via the photoacoustic effect, where optical absorption of gas molecules modulates the phase of a probe beam propagating in the fiber. By tuning the pump wavelength to a gas absorption line and the modulation frequency to match an acoustic mode, they demonstrated detection of acetylene at the parts-per-billion level. PABS provides a simple and stable setup for photoacoustic gas spectroscopy with enhanced sensitivity, and also allows characterization of the fiber microstructure by studying the acoustic resonances. Fig. 22(a) displays the scanning electron microscope (SEM) image of the anti-resonant hollow-core fiber (AR-HCF) used in the experiment. It consists of a hollow core and 7 suspended silica capillaries, with slightly different sizes. Finite element analysis shows that this structure supports several coupled acoustic modes up to a frequency range of 8 MHz. Among them, Fig. 22(b) shows a capillary mode (the first order w1 mode) with an eigenfrequency of 4.50+0.018i MHz. Fig. 22(c) shows an air mode (the first order radial $R_{01}$ mode) with an eigenfrequency of 5.15+0.70i MHz.

#### 3.2.2 Backward stimulated Brillouin scattering

When a high-intensity laser beam is incident into a fiber waveguide, it can create a periodic variation in the fiber’s refractive index due to the electrostrictive effect. This periodic variation in refractive index acts like a grating, which scatters the incident light. The scattered light, in turn, reinforces the acoustic wave that caused it, leading to a positive feedback loop. As a result, the scattered light grows exponentially, and a significant amount of energy is transferred from the incident laser beam to the scattered beam [100]. The scattered light travels in the opposite direction to the incident laser beam. Backward Brillouin scattering has found diverse applications across various domains, including sensing, signal processing, and photonic integrated circuits. Notably, in the realm of gas sensing and monitoring, it has demonstrated considerable promise and potential [92]. There are three factors related to gas properties in Backward stimulated Brillouin scattering: Brillouin frequency shift, Brillouin gain and linewidth. The frequency shift of the Brillouin frequency $\upsilon_{B}$ is proportional to optical and acoustic parameters with the following relationship: $\upsilon_{B}=2n_{eff}\nu_{a}/\lambda_{0}$, where $n_{eff}$ is the effective refractive index, $\nu_{a}$ is the sound velocity in fiber waveguide or in the gas medium, and $\lambda_{0}$ is the operating wavelength [100]. The Brillouin gain and linewidth can be calculated using the following expressions [101]:

$${\overset{\sim }{g}}_{B}=\frac{\gamma_{e}^{2}\omega^{2}}{n\nu_{a}c^{3}\rho \Gamma_{B}A_{eff}}$$

and

$$\Gamma_{B}=\frac{q^{2}}{\rho }\left(\frac{\left(\gamma -1\right)\kappa_{C}}{c_{p}}+\eta_{b}+\frac{4}{3}\eta_{s}\right)$$

where γe=∂ɛ/∂ρ is the electrostrictive coefficient in the gas medium, ω is the angular frequency of the light, ρ is the gas density, n is the gas refractive index, c is the speed of light in vacuum, and $A_{eff}$ is the acousto-optic overlap effective area. $\Gamma_{B}/2\pi$ is the spectrum linewidth which is directly proportional to the acoustic attenuation. q is the acoustic wave-vector, $\eta_{s}$ and $\eta_{b}$ are the shear and bulk viscosities, respectively, $\kappa_{C}$ is the thermal conductivity, and $c_{p}$ is the specific heat capacity at constant pressure. By monitoring the variations of Brillouin frequency shift, Brillouin gain and linewidth, the pressure of the detected gases can be measured.

In 2012, Sylvie, D. L. et al. proposed a distributed hydrogen gas sensor based on Brillouin scattering in a single-mode fiber [102]. The proposed approach is to measure the refractive index variations induced by hydrogen diffusion into the silica core based on the Brillouin Optical Time-Domain Analysis (BOTDA) system. The spatial resolution can reach 2 m over several hundred meters. The experimental results indicate that the Brillouin frequency shift depends on the hydrogen concentration in the silica core, with a shift rate of approximately 0.21 MHz/% $H_{2}$. However, the absorption of hydrogen by the silica core takes several days, rendering it impractical for real-world industrial measurements.

**Fig. 23:**
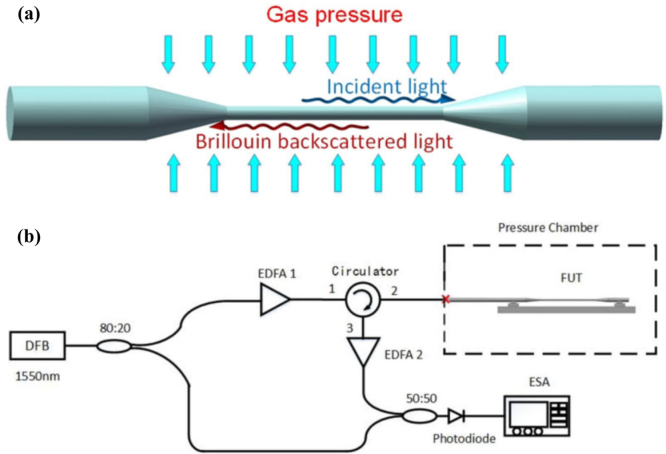
(a) The structure diagram of micro-fiber used for pressure sensing based on backward Brillouin scattering; (b) Experimental setup for backwards Brillouin spectrum measurement [103].

Backward Brillouin scattering can also be applied to measure gas pressure. In 2017, J. Huang et al. proposed a micro-fiber pressure sensor based on backward Brillouin scattering [103]. The system employs a fiber with a diameter of 1.9 μm, which is tapered from a commercial single-mode fiber. Because of the coupling between longitudinal and transverse waves resulting from the refractive index disparity between the waveguide and its surroundings, a Brillouin scattering spectrum exhibiting a multi-peak structure becomes evident. Notably, variations in ambient air pressure lead to significant shifts in the peak frequency of Brillouin scattering measured during the experiment. Fig. 23(a) shows the micro-fiber structure diagram used for pressure sensing based on backward Brillouin scattering, and Fig. 23(b) presents the experimental setup. The final experimental results indicate that the sensitivity of the sensor is approximately 0.066 MHz/kPa.

In 2021, M. Galal et al. investigated the spontaneous Brillouin scattering in a hollow-core anti-resonant fiber (HC-ARF) filled with Nitrogen ($N_{2}$) gas at different pressures [101]. A Brillouin gain of 0.029 $m^{-1}\;W^{-1}$ is achieved at a pressure of 34.7 bar. Based on this, they further explored a distributed temperature measurement performed in a nitrogen-filled AR-HCF in 2022 with the spatial resolution of about 55 cm and a temperature sensitivity of 2 MHz/K at a pressure of 34.7 bar, which is twice that of the conventional solid silica fiber sensor [104].

**Fig. 24:**
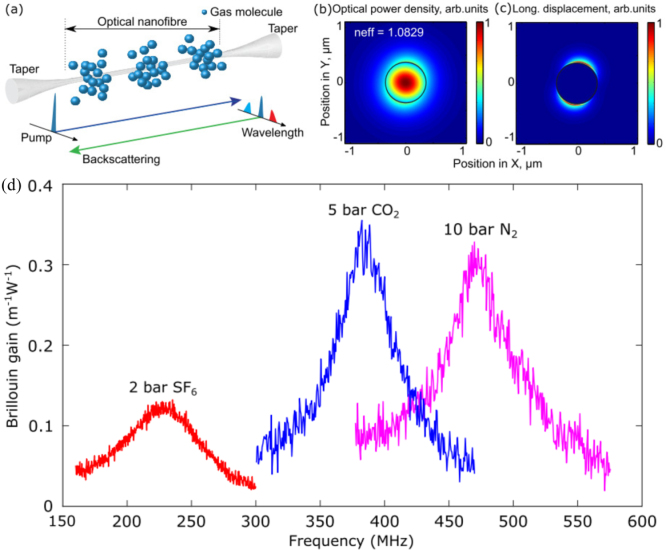
Experimental Brillouin gain spectra from the nanofibre gas cell filled with different types of gas [105].

F. Yang et al. reported the observation of strong single-pass Brillouin scattering generated by the evanescent field of a guided lightwave in pressurized carbon dioxide gas located in the immediate vicinity of a nanofiber waveguide in 2022 [105]. They demonstrated a drastic enhancement of the Brillouin scattering by increasing the gas pressure, achieving a Brillouin gain coefficient that is 11 times higher than the highest gain obtained in a hollow-core fiber and 79 times higher than in a standard single-mode fiber. Fig. 24(a) shows a conceptual diagram of the observed Brillouin scattering in the compressed gas around the optical fiber nanowire. In the optical fiber nanowire, only the forward and backward scattered photons can be guided and detected. Fig. 24(b) shows the normalized spatial power distribution of the optical modes around a 740nm diameter nanowire in 40 bar ${\mathrm{CO}}_{2}$ gas at a wavelength of 1550nm. It can be seen that due to the nanowire size being smaller than the optical wavelength, about 58% of the optical field intensity is distributed outside the nanowire, which can interact with the surrounding gas or other fluid materials. Fig. 24(c) shows the normalized longitudinal displacement of the elastic wave in the surrounding gas. As presented in Fig. 24(d), the authors also measured Brillouin spectra for different types of gases (${\mathrm{CO}}_{2}$, ${\mathrm{SF}}_{6}$, $N_{2}$) surrounding the nanofiber.

**Fig. 25:**
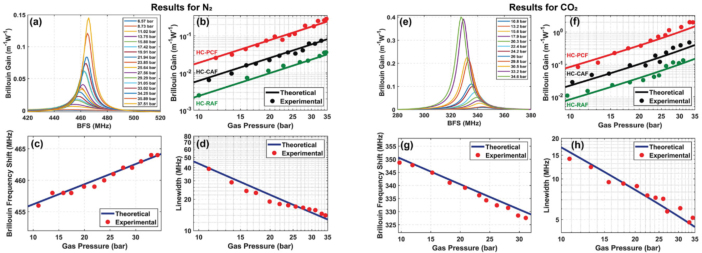
The variation of Brillouin gain and Brillouin frequency shift with gas pressure for different filling gases in anti-resonant fibers [22].

Backward Brillouin scattering in hollow-core optical fibers demonstrates unparalleled nonlinear optical amplification, surpassing the maximum nonlinear gain of standard silica single-mode fiber (SMF) by a factor of 6. In 2023, M. Galal et al. investigated Backward Brillouin Scattering gain characteristics in anti-resonant hollow-core optical fibers filled with $N_{2}$ and ${\mathrm{CO}}_{2}$ [22]. The interaction between light and sound has been demonstrated in three distinct types of gas-filled hollow-core optical fibers: a bandgap hollow-core photonic crystal fiber and two anti-resonant hollow-core fibers. When employing a conjoined-tube anti-resonant fiber, it was found that the Brillouin gain exceeded that of standard silica fibers and was substantially greater than the highest reported Raman gain in a gas-filled photonic crystal fiber. The experimental measurement results are presented in Fig. 25, which depicts the variation of Brillouin gain and Brillouin frequency shift with gas pressure for different filling gases. The figure clearly shows that the Brillouin gain increases as the gas pressure rises. Furthermore, when compared to fibers filled with $N_{2}$, the gain observed in optical fibers filled with ${\mathrm{CO}}_{2}$ is significantly higher.

**Fig. 26:**
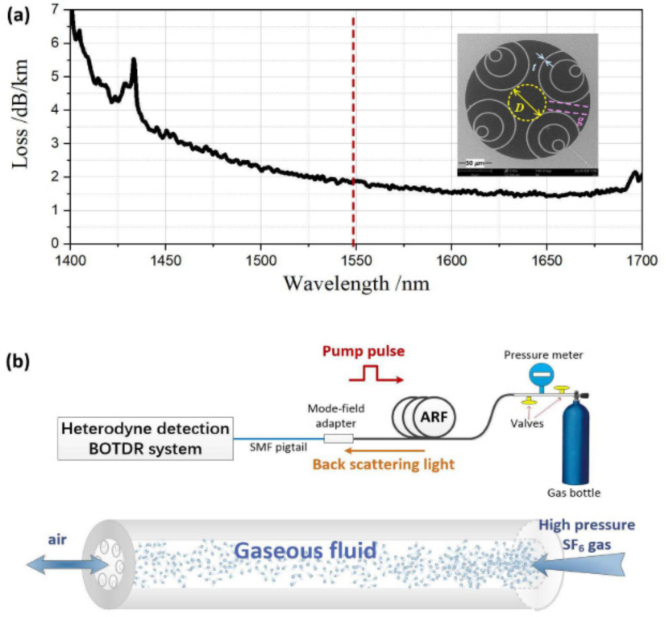
(a) Measured spectral loss of the anti-resonant hollow-core fiber along with the scanning electron microscope (SEM) image of its cross-section; (b) Schematic of RBS spectrum measurement [106].

In 2024, H. Liang et al. reported a distributed measurement method for backward Rayleigh-Brillouin scattering (RBS) spectra based on ${\mathrm{SF}}_{6}$-flowing anti-resonant hollow-core fiber using Brillouin optical time-domain reflectometry (BOTDR) technology [106]. The results demonstrated considerable variations in spectral profiles in response to alterations in gas species and pressure, thereby facilitating the unambiguous identification of ${\mathrm{SF}}_{6}$ gas amidst residual gases within the fiber. Fig. 26(a) shows the spectral loss of the anti-resonant hollow-core fiber. Fig. 26(b) shows a measurement device based on BOTDR system, used to collect RBS spectra of the anti-resonant hollow-core fiber with a length of 2.3 km. ${\mathrm{SF}}_{6}$ is used for gas filling of hollow-core optical fibers, and different gas pressures can be applied according to experimental needs. The measurement results show that the gas components at different distances can be identified at the same time. When low pressure is applied, the RBS spectrum has a Gaussian shape. The frequency detuning of Brillouin scattering in this study provides valuable reference for the study of gas diffusion problems.

Table 2 summarizes the detection performance of the Brillouin scattering based-fiber gas sensors in this review.

**Table 2:** The detection performance of the Brillouin scattering based-fiber gas sensors in this review. HC-PCF: Hollow-core photonic crystal fiber; HC-ARF: Hollow-core anti-resonant fiber.

Technique	Ref.	Target gas	Fiber type	Measurand	Performance
BSBS	[103]	Air	Microfiber	Pressure	0.066 MHz/kpa
	[105]	${\mathrm{CO}}_{2}$	Nanofibre	Pressure	1 MHz/bar
	[22]	$N_{2}$	HC-ARF	Temperature	2.18 MHz/K
		${\mathrm{CO}}_{2}$		Temperature	1.05 MHz/K
	[106]	${\mathrm{SF}}_{6}$	HC-ARF	Pressure	–

FSBS	[97]	${\mathrm{NH}}_{3}$	Tapered fiber	Concentration	1 ppb
	[16]	$H_{2}$	HC-PCF	Concentration	4260 ppm
	[99]	$C_{2}H_{2}$	HC-ARF	Concentration	8 ppb

### 3.3 Fiber gas sensing based on light-induced thermoelastic spectroscopy

**Fig. 27:**
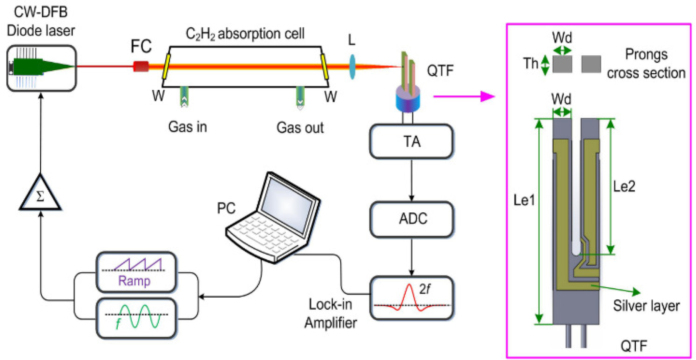
The principle of the LITES technique [111].

The fiber-based QEPAS and CEPAS technologies, introduced in the previous sections, are extensively utilized for detecting a diverse range of gases owing to their exceptional sensitivity and compact design [107], [108]. However, the necessity of placing the QTF within the gas analyte in QEPAS and CEPAS sensors restricts their applicability in specific domains [109], [110]. In 2018, Y. Ma et al. were the first to report a quartz-tuning-fork enhanced photothermal spectroscopy for $C_{2}H_{2}$ detection, which is also referred to as light-induced thermoelastic spectroscopy (LITES) [111]. The fundamental principle of LITES is based on the interaction between laser light and gas molecules, followed by the conversion of absorbed light energy into mechanical vibration through the thermoelastic effect of QTF. When a laser beam is focused on the surface of a QTF, gas molecules in the path of the beam absorb the light energy, leading to local heating. This temperature increase causes the QTF to undergo mechanical vibrations due to the thermoelastic effect. These vibrations can be detected and analyzed to determine the concentration of the target gas. LITES is a special light-induced acoustic technique because it relies on the interaction between laser light and gas molecules to generate heat and then the acoustic signals through the thermoelastic effect. LITES can eliminate the necessity of direct contact with the measured gases, which is a limitation of QEPAS. Additionally, it possesses superior anti-interference capabilities, thereby achieving even better detection sensitivity. Fig. 27 shows the principle of the LITES techniques, using an external QTF.

#### 3.3.1 SMF/MMF based LITES

In 2021, K. Zheng et al. introduced a fiber-coupled off-axis cavity-enhanced LITES sensor system for ammonia leak monitoring [112]. An optical fiber setup, incorporating 100-meter-long single-mode and 100-meter-long multi-mode fibers, is utilized for long-distance and multipoint sensing applications. In January 2022, X. Liu et al. proposed a fiber-coupled multipass-cell LITES sensor for carbon monoxide detection [113]. A fiber-coupled multipass cell with an optical path length of 40 m is incorporated into the LITES system to increase the absorption length with the advantages such as interference elimination, simplified optical alignment, and enhanced system robustness.

**Fig. 28:**
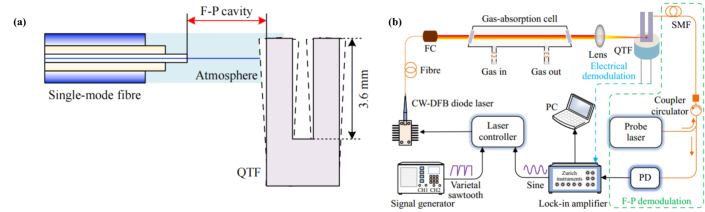
(a) Configuration of FP cavity; (b) Diagram of experimental setup of FPI-based phase demodulation of heterodyne LITES [114].

In 2023, Z. Lang et al. proposed a method for Fabry–Perot (FP)-based phase demodulation of heterodyne LITES, which uses an FP interferometer to detect QTF vibrations instead of traditional electrical signals, thus avoiding thermal noise issues [114]. The FP cavity was formed by a single-mode fiber (SMF) end face and a side of a prong of the QTF. Fig. 28(a) shows the configuration of the FP cavity. When the QTF vibrates, it causes variations in the length of the FP cavity, leading to alterations in the FPI phase. In contrast to the intensity demodulation method, the phase shift of the FPI is solely influenced by minute vibrations, and the sensitivity of this technique remains unaffected by the wavelength and power of the laser being detected. The experimental setup is shown in Fig. 28(b).

In July 2024, Y. Ma et al. proposed a high-sensitivity carbon dioxide (${\mathrm{CO}}_{2}$) sensor based on LITES technology [115]. A multipass cell (MPC) coupled with optical fibers is adopted. The laser beam is incident into the MPC through one optical fiber and then output through another optical fiber. The integration of MPC with optical fibers simplifies light coupling and resolves the optical alignment challenges typically encountered in conventional MPC systems. The experimental results show that the sensor exhibits excellent linear response to ${\mathrm{CO}}_{2}$ concentration, with a minimum detection limit of 445.91 ppm, which can be further increased to 47.70 ppm after a longer integration time of 500 s.

#### 3.3.2 HC-PCF based LITES

**Fig. 29:**
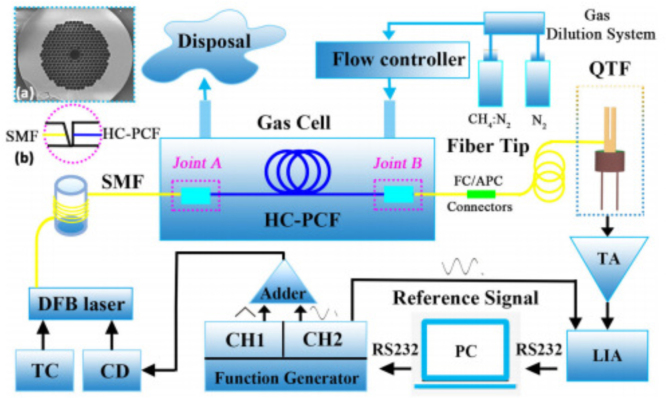
Experimental setup of the compact all-fiber LITES gas sensor [116].

Optical fiber boasts the advantages of corrosion resistance, robust insulation, and immunity to electromagnetic interference, making the application of optical fibers in LITES sensors significantly enhance their performance and practical utility [113]. In order to further improve the detection performance of fiber-based LITES sensors and minimize mode interference noise, in 2020, L. Hu et al. introduced a compact all-fiber LITES gas sensor based on a hollow-core photonic crystal fiber (HC-PCF) [116]. Fig. 29 shows the experimental setup of the compact all-fiber LITES gas sensor. A HC-PCF served as a dual-function component, acting as both a waveguide and a microcapillary gas cell. Additionally, the tip of a single-mode fiber (SMF) was utilized to direct light onto the surface of the quartz tuning fork (QTF). When compared to the free-space LITES sensor reported in the literature [111], the detection performance of the all-fiber LITES sensor based on the HC-PCF has been enhanced by approximately 10 times. However, the detection performance of multi-mode HC-PCF in this LITES gas sensor is limited by mode interference.

#### 3.3.3 HC-ARF based LITES

In 2022, Y. MA et al. proposed a compact LITES sensor based on hollow-core anti-resonant fiber (HC-ARF) [32]. HC-ARF boasts notable advantages, including near-single-mode transmission, minimal optical loss, and an expansive spectral range. It functions dually as an optical medium and a gas cell. Furthermore, HC-ARF effectively mitigates mode interference between core and other cladding modes, while also offering the benefits of a smaller footprint and simplified optical alignment. This sensor uses QTF as a thermoelastic detector for acetylene ($C_{2}H_{2}$) and carbon monoxide (CO) gases. The LITES sensor based on HC-ARF exhibits excellent linear response to analyte concentration, with the MDL for $C_{2}H_{2}$ and CO being 4.75 ppm and 1704 ppm, respectively. By using longer HC-ARF or absorption lines with stronger absorption intensity, performance can be further improved.

**Fig. 30:**
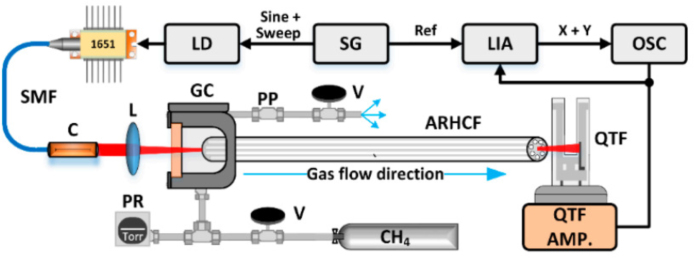
Schematic diagram of HC-ARF-LITES sensing system [40].

In 2022, P. Bojes et al. proposed a LITES configuration using hollow-core anti-resonant fiber (HC-ARF) as the gas absorption cell and QTF as the detector [40]. Combining the advantages of the high Q-factor of QTF, this sensor is used for wavelength modulation spectroscopy to detect methane (${\mathrm{CH}}_{4}$) using second harmonic signal analysis. The experimental setup is shown in Fig. 30, which achieved NNEA of $1.34\;\times \;10^{-10}$ and $2.04\;\times \;10^{-11}$ $W\;{\mathrm{cm}}^{-1}\;{\mathrm{Hz}}^{-1/2}$ in integration times of 1 and 100 s, respectively. The results demonstrate that HC-ARF offers an extended optical path while maintaining a compact and streamlined design. Meanwhile, the QTF exhibits high-frequency selectivity and facilitates low-cost detection, thereby underscoring the significant potential for optimizing costs and achieving miniaturization of gas detectors through this methodology.

**Fig. 31:**
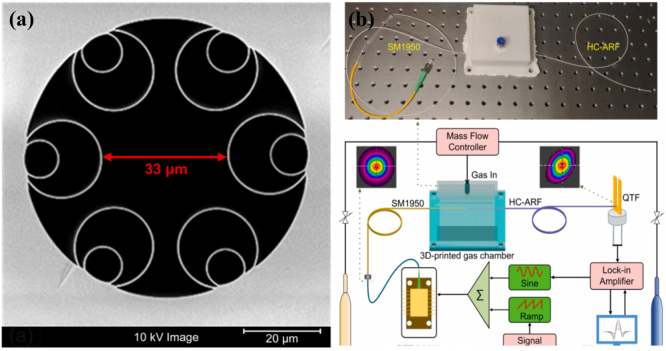
(a) Cross section image of the used HC-ARF captured by the scanning electron microscope; (b) The configuration of all-fiber LITES sensor based on HC-ARF [117].

In 2024, W. Chen et al. reported a mid-infrared all-fiber LITES sensor based on a hollow-core anti-resonant fiber (HC-ARF) [117]. A HC-ARF with a length of 55 cm was utilized. Fig. 31(a) depicts the cross-sectional image of the HC-ARF. The HC-ARF features an air core structure, comprising six sets of discrete or nested capillaries that form a negatively curved core wall. This double-layer design serves to minimize transmission loss within the fiber. The thickness of the cladding capillary walls determines the wavelength order, effectively mitigating the coupling between the core and cladding modes. Consequently, the majority of light is confined to propagate within the air core. Fig. 31(b) shows the experimental setup. The experimental results show that the HC-ARF based all fiber LITES sensor has a good linear response to CO concentration.

Table 3 summarizes the detection performance of the fiber gas sensors based on LITES in this review.

**Table 3:** The detection performance of the fiber gas sensors based on LITES in this review. MDL: minimum detection limit; NNEA: normalized noise equivalent absorption coefficient; HC-PCF: Hollow-core photonic crystal fiber; HC-ARF: Hollow-core anti-resonant fiber.

Sensor type	Target gas	Fiber type	Effective optical	MDL	Integ.time	NNEA
			PathLength (m)		(s)	cm${}^{-1}$ W/Hz${}^{1/2}$
Conventional	$C_{2}H_{2}$	/	0.2	718 ppb	1	$7.63\times 10^{-9}$
LITES [111]						
Fiber-based	${\mathrm{NH}}_{3}$	SMF	∼9.28	8.5 ppm	0.1	$1.7\times 10^{-9}$
LITES [112]						
Fiber-based	$C_{2}H_{2}$	SMF	0.2	/	/	/
LITES [114]						
Fiber-based	${\mathrm{CO}}_{2}$	SMF	40	47.70 ppm	500	/
LITES [115]						
Fiber-based	${\mathrm{CH}}_{4}$	HC-PCF	∼0.3	/	0.3	$9.66\times 10^{-9}$
LITES [116]						
Fiber-based	CO	HC-ARF	0.75	1704 ppm	200	/
LITES [32]	$C_{2}H_{2}$	HC-ARF	0.75	4.75 ppm	200	/
Fiber-based	${\mathrm{CH}}_{4}$	HC-ARF	0.95	220 ppbv	100	$2.04\times 10^{-11}$
LITES [40]						
Fiber-based	CO	HC-ARF	0.55	3.85 ppm	100	$6.32\times 10^{-8}$
LITES [117]						

## 4 Conclusion and discussion

Fiber-based gas sensing technology, leveraging light-induced acoustic/elastic techniques, has emerged as a promising contender to traditional bulk spectrometer sensing, distinguished by its compact footprint, remote sensing capabilities, and multiplexing potential. However, it confronts several hurdles in fulfilling the genuine requirements of gas sensing applications: (1) Fiber-based gas sensors, adept at detecting gases at low concentrations, often grapple with selectivity. Unlike bulk spectrometer sensing, which differentiates gases with high precision, fiber-based sensors might necessitate supplementary signal processing or the employment of specific materials to attain comparable selectivity levels. (2) Bulk spectrometer sensing boasts a broader measurement and dynamic range, facilitating the detection of gases across a wide concentration spectrum, from very low to very high levels. Conversely, fiber-based gas sensors may exhibit limited measurement ranges, potentially hindering their application in specific scenarios. (3) Bulk spectrometer systems are generally more resilient and less influenced by environmental variables like temperature fluctuations, humidity, and vibrations. Fiber-based gas sensors, particularly those relying on evanescent wave sensing, may be more susceptible to these factors, potentially compromising their performance and reliability. (4) Integrating fiber-based gas sensors into existing systems can be challenging due to their specialized nature and the need for specialized optical components. In contrast, bulk spectrometer sensing systems are often more modular and easier to integrate into various applications. Additionally, scaling up fiber-based gas sensing systems to meet the demands of large-scale gas monitoring networks can be difficult and costly.

In the future, in our opinion, the development direction of optical fiber-based gas sensors that utilize light-induced acoustic/elastic techniques can be summarized as follows: (1) Continue to explore the application of technologies such as fiber-based photoacoustic spectroscopy, Brillouin scattering, and photothermal elastic spectroscopy in the field of gas sensing, further improving the detection sensitivity and selectivity. (2) Develop new fiber structures to enhance light-gas interaction and improve gas sensing performance, such as hollow-core photonic crystal fibers and anti-resonant hollow-core fibers. (3) Study the application of micro/nano fibers in gas sensing, utilizing their unique waveguide characteristics to achieve higher sensitivity and smaller volume sensors. (4) Further optimize the integration and stability of the fiber-optic gas sensing system, realizing more compact, stable, and efficient all-fiber gas sensing devices. (5) Explore the potential of fiber-optic gas sensing technology in practical applications, such as environmental monitoring, industrial process control, and medical diagnosis.

In conclusion, we reviewed the recent developments in optical fiber-based gas sensors that utilize light-induced acoustic/elastic techniques. The key techniques discussed are photoacoustic spectroscopy, Brillouin scattering, and light-induced thermoelastic spectroscopy. The advantages of using optical fibers for gas sensing are highlighted, such as high detection sensitivity, resistance to electromagnetic interference, fast detection speed, and portability. Different types of optical fibers used for gas sensing are also introduced, including hollow-core fibers, photonic crystal fibers, and micro/nano fibers, and their unique properties and applications are discussed. While fiber-based gas sensing technology faces challenges in meeting the true needs of gas sensing applications, ongoing research and development efforts are paving the way for future advancements. By addressing these challenges and exploring new directions, fiber-based gas sensing technology has the potential to revolutionize the field of gas detection and monitoring.

## CRediT authorship contribution statement

**Yuhui Liu:** Writing – original draft, Investigation. **Yue Qi:** Writing – original draft, Investigation. **Yangjian Cai:** Writing – review & editing, Supervision. **Xiaoyi Bao:** Writing – review & editing, Supervision. **Song Gao:** Writing – review & editing, Writing – original draft, Supervision, Conceptualization.

## Declaration of competing interest

The authors declare that they have no known competing financial interests or personal relationships that could have appeared to influence the work reported in this paper.

## Data Availability

Data will be made available on request.
